# Clinician perceptions on barriers and facilitators to 1‐year surveillance colonoscopy completion in survivors of colorectal cancer

**DOI:** 10.1002/cam4.70244

**Published:** 2024-09-24

**Authors:** C. Natasha Kwendakwema, Talor Hopkins, Ari Bell‐Brown, Vlad V. Simianu, Veena Shankaran, Rachel B. Issaka

**Affiliations:** ^1^ Hutchinson Institute for Cancer Outcomes Research Fred Hutch Cancer Center Seattle Washington USA; ^2^ Center for Digestive Health Virginia Mason Franciscan Health Seattle Washington USA; ^3^ Public Health Sciences Division Fred Hutch Cancer Center Seattle Washington USA; ^4^ Division of Gastroenterology University of Washington School of Medicine Seattle Washington USA

**Keywords:** barriers, colonoscopy, colorectal cancer, facilitators, surveillance

## Abstract

**Introduction:**

Colorectal cancer (CRC) is the second leading cause of cancer deaths in the United States. Surveillance colonoscopy is recommended 1‐year after surgical resection for patients with stage I‐III CRC; however, only 18%–61% of CRC survivors complete this test. This study describes clinician‐identified barriers and facilitators to surveillance colonoscopy among CRC survivors.

**Methods:**

We conducted semi‐structured interviews with clinicians until thematic saturation was achieved. Interviews were analyzed using the social cognitive theory.

**Results:**

Thirteen clinicians were interviewed, and all identified health system‐level barriers to surveillance colonoscopy completion; the most common being fragmented care due to patients receiving care across many health systems. Clinicians also identified social determinants of health barriers (e.g., geographical distance between patients and health systems) to 1‐year surveillance colonoscopy completion.

**Conclusions:**

Clinicians identified several potentially modifiable barriers to 1‐year surveillance colonoscopy completion which, if addressed, could improve post‐treatment care and outcomes among stage I‐III CRC survivors.

## 1 INTRODUCTION

Colorectal cancer (CRC) is the second leading cause of cancer deaths in the United States.^1^ The standard of care for stage I‐III CRC includes surgical resection followed by adjuvant chemotherapy for some high‐risk stage II and for all stage III CRC patients.^2^ Since patients with stage I‐III CRC have a higher risk for recurrence and developing metachronous colorectal tumors, national guidelines recommend a surveillance colonoscopy 1 year after surgical resection; yet, 1‐year surveillance colonoscopy completion rates remain suboptimal.^2^, ^3^, ^4^, ^5^, ^6^, ^7^ Survivors of CRC are at high risk of local recurrence, so understanding the potentially modifiable barriers and facilitators to surveillance colonoscopy completion is critical to improving care outcomes.

Few studies have evaluated the barriers and facilitators to surveillance colonoscopy completion in CRC survivors.^8^, ^9^ Our prior analysis found that older age, higher stage CRC, and living without a partner were associated with lower adherence to surveillance colonoscopy.^10^ Other factors such as distance from an endoscopy suite also impact adherence.^11^ Many demographic and clinical factors identified to date are non‐modifiable, and administrative claims data may not completely capture intervenable factors. Qualitative studies could fill the knowledge gap and inform interventions to address this issue. This study aimed to describe clinician‐identified barriers and facilitators to 1‐year surveillance colonoscopy completion.

## 2 METHODS

We conducted semi‐structured interviews of clinicians caring for CRC patients. We adhered to the Consolidated Criteria for Reporting Qualitative Research (COREQ) reporting guideline and the US Federal Policy for the Protection of Human Subjects.^12^ The study was approved by the Fred Hutchinson Cancer Center/University of Washington Cancer Consortium's Institutional Review Board. All participants provided consent and received a $100.00 cash incentive.

### 2.1 Study setting, sampling and recruitment

Clinicians were recruited via email from the Hutchinson Institute for Cancer Outcomes Research (HICOR) Value in Cancer Care (VCC) network, an academic community‐integrated consortium of 217 medical oncologists from 14 health systems in Washington state (Data S1). Clinicians included medical oncologists, colorectal surgeons, gastroenterologists, and advanced practice providers (APPs) who were VCC clinic employees and ≥18 years old. Participants were recruited from two clinics with higher surveillance colonoscopy completion rates and one clinic with lower surveillance colonoscopy completion rates based on the 2021 HICOR Annual Quality Report and prior research.^10^, ^13^

### 2.2 Interview guide development

We conducted grounded interviews using open‐ended questions to explore beliefs and knowledge about surveillance colonoscopies.^14^ The semi‐structured interview guide was developed by the study team and informed by the Health Belief Model (HBM).^15^ The HBM was developed to inform behaviors related to uptake of health services and was selected because of its extensive use in understanding barriers to CRC screening (Data S2).

### 2.3 Data collection

Semi‐structured interviews and demographic surveys were completed via a video‐conferencing platform by authors T.H and C.N.K. Interviews were recorded, deidentified, and transcribed verbatim. Following accepted standards of rigor in qualitative research, we collected data until thematic saturation was reached.^16^

### 2.4 Data analysis

Descriptive data were reported as proportions or medians and interquartile ranges (IQRs). Three authors (C.N.K., A.B.B., and R.B.I.) developed an initial set of codes inductively and deductively informed by the social cognitive theory (SCT). SCT is a health behavior theory that connects behavior to environment and was selected because it provides guidance for developing interventions to change health‐related behaviors.^17^, ^18^, ^19^ Codes and definitions were discussed and modified across the research team prior to finalization. The lead coder (C.N.K) applied the codebook across interviews to identify key themes using the Dedoose qualitative coding software version 9.0.107 (SocioCultural Research Consultants).

## 3 RESULTS

### 3.1 Clinician characteristics

Twenty‐one clinicians received an invitation to participate, 17 provided consent, and 13 completed interviews. Ten were physicians, and three were APPs (Table 1). Nine clinicians (69.2%) identified as female and 10 (76.9%) spent more than 75% of their time in direct patient care. Eight clinicians (61.5%) had a clinical practice size of greater than 100 CRC patients.

**TABLE 1:** Clinician participant characteristics.

Characteristic	Participants (%) *N* = 13
Clinician type	
Physician^a^	10 (76.9%)
Advanced practice provider	3 (23.1%)
Sex	
Women	9 (69.2%)
Men	4 (30.8%)
Race	
White	8 (61.5%)
Asian	5 (38.5%)
Ethnicity	
Non‐Hispanic	13 (100%)
Time in practice	
More than 5 years	7 (53.8%)
3–5 years	4 (30.8%)
Less than 3 years	2 (15.4%)
Clinical effort %, median (IQR)	75 (75, 100)
Clinical practice size	
Greater than 100	8 (61.5%)
50–100	1 (7.7%)
<50	4 (30.8%)

^a^ Four medical oncologists, four colorectal surgeons, and two gastroenterologists.

### 3.2 Barriers to surveillance colonoscopy completion

Clinicians identified barriers to surveillance colonoscopy completion across two main themes— (1) health system‐level barriers and (2) patient‐level barriers. Themes and quotations are summarized in Table 2.

**TABLE 2:** Clinician‐identified key themes, subthemes, and supporting quotations.

Themes and subthemes	Participants, *N* (%)	Supporting quotation
Barriers to surveillance		
Health system‐level factors		
Organizational factors		
Fragmented care	10 (76.9%)	“Yeah, just anytime we have to coordinate or refer to an outside institution, you know, it, most of the time gets done without a hitch, but sometimes it just requires [more] and it's just more moving pieces. A little bit more on the patient to make sure to receive that call from the other place, and depending on whether the other place calls them in time and communicating what needs to be done with the other institution.”
Workplace pressures	6 (46.2%)	“I mean, we know what patients should do, we know we should remind them, but we're busy, so we're distracted.”
Long wait times	6 (46.2%)	“I think one issue is that there's long lead times at many places. Patients report going, you know, requesting a colonoscopy and sometimes having a long time, many many months before they could even have a procedure.”
Staffing shortage	3 (23.1%)	“Other barriers are health system resources, so you know, we're short staffed, and so we've had to close rooms every once in a while.”
Clinician cognitive factors		
Clinician expectations	5 (38.5%)	“…so I do tend to rely on the treating physicians once there, once the patient is established with them, to refer back to us for their post‐treatment colonoscopy.”
Clinician lack of knowledge	5 (38.5%)	“A lot of time you know, when patients have more of a piecemeal care at different place, their local providers might not be that up to date in terms of the recommendation for colorectal cancer surveillance management. And that's really when things get missed.”
Patient‐level barriers		
SDOH		
Access (geography)	6 (46.2%)	“We have to rely on patients being able to come back to (name of city), which again, is transportation, overnight stay cost, et cetera, et cetera. So, it can be a lot.”
Other life obligations	5 (38.5%)	“I think just because of the time commitment, with other things that are happening in life, if patients with a history of colon cancer have other important things going on, like if they're the caregiver for somebody in their family, if they have a family member who's relying on them or are sick, or if they're one of the younger patients, if they have young kids, I think just the kind of a two‐day commitment to getting it done can definitely just be a barrier overall.”
Insurance issues	5 (38.5%)	“With insurance changing constantly, like where they accept, or like institutions changing which insurance they cover, if somebody is trying to come back to us for their scope but their insurance has changed and we're no longer covered and then they have to try and establish care, I know that that can be an issue.”
Lack of transport/lack of chaperone to procedure	4 (30.8%)	“I see a lot sometimes of needing a family member or friend to escort them to the procedure and potentially not having someone, and therefore that being a barrier to having it done.”
Patient cognitive factors		
Patient forgetting the need for surveillance colonoscopy	3 (23.1%)	“…I think one of the other barriers are that if they sort of forget, especially for stage one, because our stage one patients have no other surveillance.”
Patient expectations	2 (15.4%)	“I think some people, if they're not told to anticipate that once they've had the cancer removed, they're kind of done. But we do try to specify, at least in our clinic, that they will need much closer endoscopic surveillance.”
Negative past experience	2 (15.4%)	“It reminds them of what it felt like when they were sick, or it has them worried that there might be a finding that indicates that their disease has come back, or something concerning.”

Facilitators to surveillance		
Health system‐level factors		
Patient interaction with clinicians		
Patient reminders/education	11 (84.6%)	“If they hear it from their doctors or their APP's, I think they know it's important. So we don't just send a letter, in contrast, we say make sure you do this, you know, they hear it directly from us at a visit. And then sometimes when they raise challenges with doing bowel preps, and we can talk them through that and counsel them through, make sure they get the right prep.”
Routine interactions with healthcare system	8 (61.5%)	“We try to adhere to the guidelines regarding follow up of our patients, and frequency of, and that sort of thing. So that relationship that you have with the patient every few months and that visit is a good opportunity to reinforce the importance of it.”
Organizational factors		
Primary and specialty care integration (organizational)	6 (46.2%)	“And then I think having it be an interdisciplinary effort so it's not just the providers but the whole team is sort of aware of the importance, and then it's something that the clinical nurse can follow up with the patient as well, if they're having any delays or any questions, they have that relationship with the nurse too and they can continue the conversation.”
EHR tools	4 (30.8%)	“We have a health maintenance tab that's in our electronic medical record and it flags if someone is not up to date with their surveillance colonoscopy.”
Scheduling accommodations	4 (30.8%)	“You know, patients are coming from far away. Maybe they don't want to come over the mountains because the passes are hard to travel, so they're willing to consolidate their visits you know, that are either a little bit earlier or a little bit later.”
Cognitive factors		
Patient motivation to prevent recurrence	7 (53.8%)	“I think one is, patient wise, I think there's internal motivation, you know, they're motivated to get their checkups and know that this is part of making sure that they are clear and their cancer is not coming back. And so there's motivation on the patient's part, they have interest in it.”
Patient–clinician concordance about colonoscopy importance	3 (23.1%)	“The whole group has buy in, the medical oncologist it's part of their pathway. We just have a cohesive care plan that everyone needs to follow, this is the surveillance, we go by NCCN guidelines and everyone's on board to make sure these boxes are checked.”

Abbreviations: APP, advanced practice provider; NCCN, national comprehensive care network.

#### 3.2.1 Health system‐level barriers

##### Organizational factors

The most frequently reported organizational factors were fragmented care (10 participants [76.9%]), workplace pressures (6 participants [46.2%]), and long wait times (6 participants [46.2%]). Clinicians commented that managing CRC patients across multiple institutions with non‐integrated EHRs led to fragmented care. Workplace pressures (e.g., busy clinic schedules, competing medical issues during visits) hindered reminding patients about surveillance colonoscopy completion.

##### Clinician cognitive factors

The most reported clinician‐identified cognitive barriers were differing expectations about which specialist had ownership of scheduling or tracking surveillance procedures (5 participants [38.5%]) and clinicians' lack of knowledge about guidelines (5 participants [38.5%]). Clinicians acknowledged that relying on other colleagues like oncologists or primary care doctors to remind patients about their surveillance likely contributed to missed follow‐up. Limited exposure to CRC patients among some clinicians also led to a lack of knowledge of surveillance requirements.

#### 3.2.2 Patient‐level barriers

##### Social determinants of health

The most common patient‐level SDOH barriers were geography (i.e., distance from home to the healthcare facility; 6 participants [46.2%]), health insurance (5 participants [38.5%]), other life obligations (5 participants [38.5%]), and lack of procedural transportation or a chaperone (4 participants [30.8%]). Clinicians commented that patients that lived farther from the health system faced challenges traveling for surveillance colonoscopies, especially while completing a bowel preparation. Similarly, transportation barriers and lack of a caregiver contributed to delays or missed appointments.

#### 3.2.3 Patient cognitive factors

The patient‐centered cognitive factors identified as barriers included patients forgetting that they needed a surveillance colonoscopy (3 participants [23.1%]), patients thinking that their CRC management was complete after surgical resection (2 participants [15.4%]), and patients missing or delaying surveillance colonoscopies due to negative past experiences with endoscopy (2 participants [15.4%]).

##### Facilitators to surveillance colonoscopy completion

Facilitators to surveillance colonoscopy completion fell into two main themes— (1) health system‐level facilitators and (2) patient‐cognitive factors. Themes and quotations are summarized in Table 2.

#### 3.2.4 Health system‐level facilitators

##### Patient interactions with clinicians

Facilitators to surveillance colonoscopy completion included patients receiving reminders and education from clinicians (11 participants [84.6%]) and routine clinician‐patient visits (8 participants [61.5%]). Clinicians commented that reminding patients and proactively addressing concerns led to increased colonoscopy completion rates.

##### Organizational factors

The organizational facilitators most cited by clinicians were primary and specialty care integration (6 participants [46.2%]), use of EHR tools (4 participants [30.8%]), and scheduling accommodations for patients (4 participants [30.8%]). Clinicians commented that good communication regarding CRC surveillance from specialists to primary care improved colonoscopy completion. EHR tools such as reminders also helped facilitate surveillance completion.

#### 3.2.5 Cognitive factors

The most common cognitive facilitators to colonoscopy completion were patients' motivation to prevent recurrent disease (7 participants [53.8%]) and patient–clinician concordance about the importance of surveillance colonoscopy (3 participants [23.1%]).

##### Potential systems improvements to address inadequate surveillance

Clinicians identified healthcare systems improvements that might improve surveillance colonoscopy completion, including increased use of electronic reminders (6 participants [46.2%]), advanced scheduling of colonoscopies (6 participants [42.6%]), increased use of nurse navigators (5 participants [38.5%]), and patient checklists (2 participants [15.4%]).

Clinicians suggested that integrating EHR alerts for surveillance colonoscopies, akin to cancer screening reminders, might enhance completion rates. Scheduling colonoscopies during initial post‐operative appointments, patient‐owned electronic checklists outlining surveillance needs, and nurse navigation were all identified as potential areas for intervention.

## 4 DISCUSSION

Our study identified several barriers and facilitators to 1‐year surveillance colonoscopy completion from the clinician‐perspective. All clinicians identified at least one organizational factor as a barrier to surveillance colonoscopy completion, but some organizational factors when present (e.g., primary and specialty care integration), facilitated surveillance colonoscopy completion. Ultimately, clinicians identified several areas for potential intervention to improve 1‐year surveillance colonoscopy completion among CRC survivors. To our knowledge, our study is the first to examine clinician perspectives on 1‐year surveillance colonoscopy completion.

Our qualitative study provides additional evidence to support some previously reported barriers. For example, one study found that inadequate insurance was associated with lower surveillance colonoscopy completion, which is concordant with our study.^20^ Given the limited number of studies looking at surveillance colonoscopy barriers in CRC survivors, screening studies could serve as a proxy for identifying potential barriers. Screening studies have identified transportation issues and inflexible work schedules as barriers to colonoscopy completion.^21^, ^22^ Our study suggests that similar barriers are encountered among CRC survivors who require surveillance colonoscopies.

Clinicians suggested several facilitators to 1‐year surveillance colonoscopy completion including routine clinician–patient interactions. Previous quantitative studies have shown that patients who see their PCPs or an oncologist within the first year of a CRC diagnosis are more likely to receive surveillance colonoscopies.^23^, ^24^ Similarly, physician endorsement is positively associated with obtaining a colonoscopy in the non‐CRC population.^25^ Clinicians offered several potentially modifiable intervention targets to increase 1‐year surveillance colonoscopy completion including electronic reminders for clinicians.

## 5 STRENGTHS AND LIMITATIONS

The strengths of this study are its qualitative study design, which allowed for detailed inquiry not otherwise possible through EHR review and its diverse participant population making our results generalizable to clinicians of varying specialties and practice scopes. A limitation of our study was the small clinician sample size from a limited number of regional clinics.

## 6 CONCLUSION

Understanding clinician perspectives is important for the development of effective interventions to improve surveillance colonoscopy completion rates and has the potential to improve outcomes for CRC survivors.

## AUTHOR CONTRIBUTIONS

**C. Natasha Kwendakwema:** Formal analysis (equal); investigation (equal); writing – original draft (lead); writing – review and editing (equal). **Talor Hopkins:** Investigation (equal); project administration (equal); writing – review and editing (equal). **Ari Bell‐Brown:** Data curation (equal); formal analysis (equal); project administration (equal); writing – review and editing (equal). **Vlad V. Simianu:** Project administration (equal); writing – review and editing (equal). **Veena Shankaran:** Methodology (supporting); writing – review and editing (equal). **Rachel B. Issaka:** Conceptualization (lead); data curation (lead); formal analysis (supporting); funding acquisition (lead); investigation (lead); resources (equal); supervision (lead); writing – original draft (supporting); writing – review and editing (lead).

## FUNDING INFORMATION

Research reported in this publication was supported by the National Cancer Institute of the National Institutes of Health under award numbers T32CA009515 (C.N.K.), K08CA241296 (R.B.I.), and P30 CA015704 (R.B.I.).

## CONFLICT OF INTEREST STATEMENT

Dr. Issaka has received consulting fees from Guardant Health, Inc., outside the submitted work. No other conflicts of interest were reported from the authors.

## ETHICS STATEMENT

This study was performed in accordance with the Declaration of Helsinki and was approved by the Fred Hutchinson Cancer Center/University of Washington Cancer Consortium's Institutional Review Board.

## DISCLAIMER

The content is solely the responsibility of the authors and does not necessarily represent the official views of the National Institutes of Health. The funder had no role in the design and conduct of the study; collection, management, analysis, and interpretation of the data; preparation, review, or approval of the manuscript; and decision to submit the manuscript for publication.

## Supporting information


Data S1.

Data S2.

## Data Availability

The data to support the findings of this study without personal identifiers may be made available upon request from corresponding author C.N.K.
