# Supplementary material for: Clinician perceptions on barriers and facilitators to 1‐year surveillance colonoscopy completion in survivors of colorectal cancer
Source: Cancer Med. 2024 Sep 24;13(18):e70244. doi: 10.1002/cam4.70244 (PMC11420656; doi:10.1002/cam4.70244)
Supplement: Supplementary file 1 — Data S1. [file CAM4-13-e70244-s002.docx]

**Provider Interview Guide**

**Barriers to 1-year Surveillance Colonoscopy Project**

Hello [**participant name**],

My name is [**interviewer name**] and I am calling today to talk about surveillance colonoscopies that are recommended to be completed approximately 1 year after colorectal cancer surgery. Is now still a good time to complete this interview?

As we discussed during the consent, this interview will take approximately 30 minutes and your participation is voluntary. You can choose not to answer any of the questions I ask. If you would like to skip a question, just say “skip” and I will move on to the next question.

**[TURN RECORDER ON]** I’m starting the recording. Is this ok with you?

***Grounded probes/prompts:*** *If responses are limited or require clarification, probes may be used to elicit more detailed responses. Probes should use words or phrases presented by the participant using one of the following formats:*

***1. What do you mean by ____________ ?***

***2. Tell me more about  ____________.***

***3. Can you give me an example of ____________ ?***

***4. Can you tell me about a time when ____________ ?***

***5. Who ____________ ?***

***6. When ____________ ?***

1. In what capacity do you interact with patients with stage I-III colorectal cancer? **[Knowledge]**
2. What is your understanding of the current recommendations for surveillance colonoscopy after stage I-III colorectal cancer treatment? **[Knowledge]**
3. In your own words, please tell me what you think patients who have had colon cancer know about colonoscopy after treatment? **[Knowledge]**
4. Do you or someone else from your healthcare team talk to patients about getting a colonoscopy after colon cancer treatment? **[Knowledge]**

**(If yes):**

- 1. What do you or people from your team tell patients about colonoscopy after surgery for colon cancer? **[Knowledge]**
  2. Within what time frame do you or people from your team tell patients about colonoscopy after surgery for colon cancer? **[Knowledge]**

1. What are some potential barriers that patients encounter when completing a colonoscopy after colon cancer treatment? **[Barriers]**
   1. What are the health system factors that create barriers to surveillance colonoscopy? **[Health System Barriers]**
2. What are some potential facilitators that patients experience when completing a colonoscopy after colon cancer treatment? **[Facilitators]**
   1. What are the health system factors that facilitate surveillance colonoscopy completion for patients? **[Health System Facilitators]**
3. What could clinical providers do to help patients who need a colonoscopy after colorectal cancer surgery? **[Facilitators]**
4. What strategies are in place (if any) to coordinate surveillance colonoscopy completion?  **[Health System]**
5. How well do you feel these strategies work to ensure follow-up colonoscopy completion? **[Health System]**
6. How does your clinic or health system keep track of patients who need a surveillance colonoscopy? [**Health System]**
7. If you oversaw surveillance colonoscopy completion in CRC survivors in your clinic, what would you change to improve follow-up? [**Health System]**

1. Do you have any questions for us, or is there anything else you would like us to know?

Thank you for completing the interview. We now have some additional questions we would like to ask as well, and I will be turning off the recorder for this part of the interview

**[TURN RECORDER OFF]**

**Demographics Questions:**

1. What is your race? (Choose the one you most identify with) **[Race]**
2. Black / African-American
3. Asian / Asian-American
4. White / Caucasian
5. American Indian / Native-American
6. Pacific Islander (Hawaii, Guam, Samoa, Other South Pacific Island)
7. Mixed (Bi- or Multi-Racial) – Specify _________________
8. Other – Specify _____________________
9. What is your ethnicity? (Choose the one you most identify with) **[Ethnicity]**
10. Hispanic / Latino
11. Non-Hispanic
12. What is your sex?
    1. Male
    2. Female
    3. Other
13. What is your role in the clinic?  **[Job Responsibility]**
14. Physician
15. Nurse
16. Advanced practice practitioner (Physician Assistant or Nurse Practitioner)
17. Other - Specify
18. How much of your time do you spend seeing patients? **[Job Responsibility]**
    1. 100%
    2. 75%
    3. 50%
    4. 25%
    5. Other- Specify
19. How long have you worked at this practice? **[Job Responsibility]**
    1. Less than a year
    2. 1 to 2 years
    3. 3 to 5 years
    4. >5 years
20. What is your clinical practice size (referring to total number of patients on your patient panel who have colon cancer)? **[Job Responsibility]**
    1. 0 to 10
    2. 10-30
    3. 30-50
    4. 50-100
    5. >100
