# Supplementary figures and images for: Clinician perceptions on barriers and facilitators to 1‐year surveillance colonoscopy completion in survivors of colorectal cancer
Source: Cancer Med. 2024 Sep 24;13(18):e70244. doi: 10.1002/cam4.70244 (PMC11420656; doi:10.1002/cam4.70244)

**Supplemental Material 1**: Value in Cancer Care (VCC) Network Map


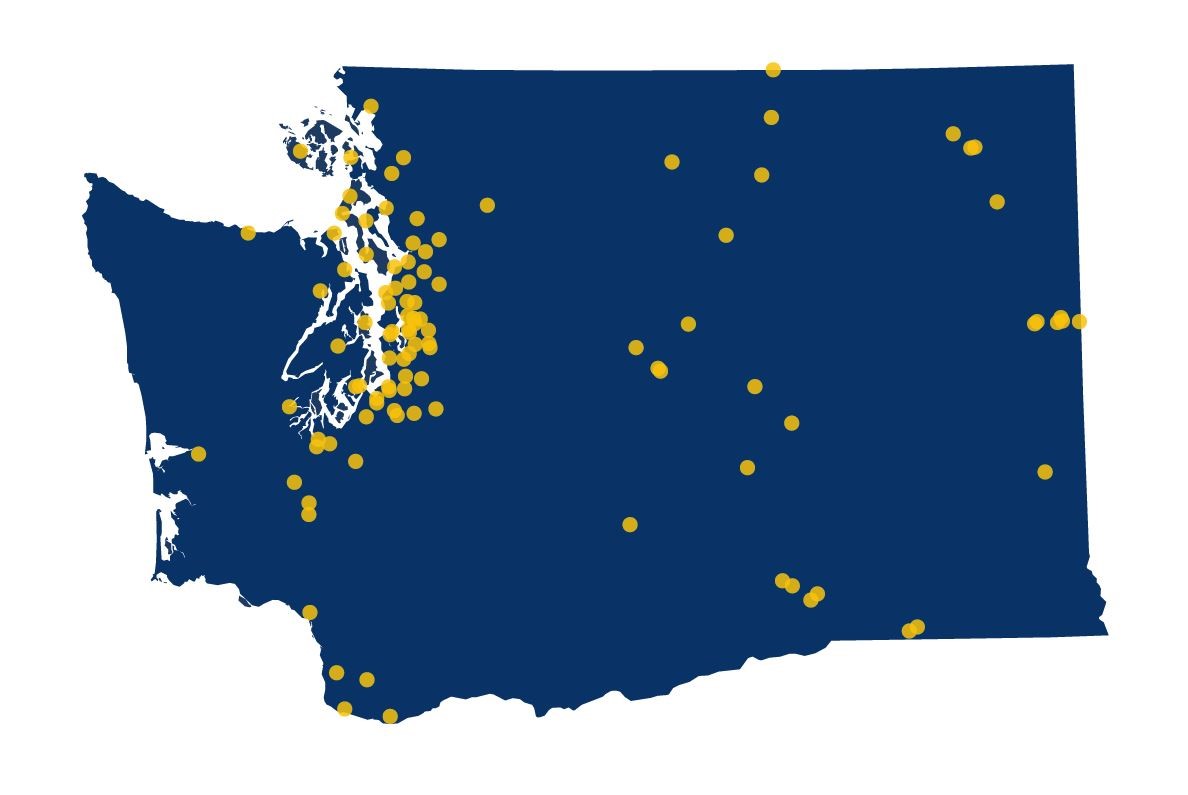

Supplement: Supplementary file 2 — Data S2. [file CAM4-13-e70244-s001.docx]
